# Synergistic tumor inhibition of colon cancer cells by nitazoxanide and obeticholic acid, a farnesoid X receptor ligand

**DOI:** 10.1038/s41417-020-00239-8

**Published:** 2020-10-13

**Authors:** Junhui Yu, Kui Yang, Jianbao Zheng, Wei Zhao, Xuejun Sun

**Affiliations:** Department of General Surgery, First Affiliated Hospital of Xi’an Jiaotong University, 277 West Yanta Road, 710061 Xi’an, China

**Keywords:** Colorectal cancer, Targeted therapies

## Abstract

The tumor-suppressive role of Farnesoid X receptor (FXR) in colorectal tumorigenesis supports restoring FXR expression as a novel therapeutic strategy. However, the complicated signaling network and tumor heterogeneity hinder the effectiveness of FXR agonists in the clinical setting. These difficulties highlight the importance of identifying drug combinations with potency and specificity to enhance the antitumor effects of FXR agonists. In this study, we found that the β-catenin level affected the antitumor effects of the FXR agonist OCA on colon cancer cells. Mechanistic studies identified a novel FXR/β-catenin complex in colon cancer cells. Furthermore, the depletion of β-catenin expedited FXR nuclear localization and enhanced its occupancy of the SHP promoter and thereby sensitized colon cancer cells to OCA. Furthermore, we utilized a drug combination study and identified that the antiparasitic drug nitazoxanide (NTZ) abrogated β-catenin expression and acted synergistically with OCA in colon cancer cells. The combination of OCA plus NTZ exerts synergistic tumor inhibition in CRC both in vitro and in vivo by cooperatively upregulating SHP expression. In conclusion, our study offers useful evidence for the clinical use of FXR agonists combined with β-catenin inhibitors in combating CRC.

## Introduction

Colorectal cancer (CRC) is the second most common cause of cancer-related death [1]. Globally, approximately 1,800,000 new cases are diagnosed as CRC every year. Due to distant metastasis and relapse, most advanced-stage of CRC has a poor prognosis [2, 3]. The five-year survival rate of stage I CRC patients exceeds 90%; however, the five-year survival rate of stage IV CRC patients is slightly higher than 10% [4]. An increasing number of genetic and molecular alterations have been recognized in colorectal carcinogenesis, including genetic mutations, microsatellite instability, and DNA hypermethylation [5].

Canonical Wnt/β-catenin signaling plays a vital role in colorectal tumorigenesis [6]. Persistent activation of Wnt signaling is characterized by nuclear accumulation of β-catenin [7]. β-Catenin can be stabilized in approximately 75% of CRC patients by inactivating mutations in APC, and in an additional 5% of patients by phosphodegron mutations in β-catenin [8]. These mutations facilitate the occurrence of microadenoma, whereas other mutations, including KRAS, p53, and SMAD4 mutations, contribute to a malignant transition from microadenoma to larger adenomas and adenocarcinomas [9]. Targeting the Wnt/β-catenin pathway is supposed to be a rational therapeutic strategy for CRC [10]. However, to date, no anticancer drugs targeting this signaling pathway have advanced to clinical applications. Porcupine inhibitors and tankyrase inhibitors exert inhibitory effects on Wnt signaling upstream of the β-catenin destruction complex [11], and are therefore unlikely to effectively fight against β-catenin in CRC with the most frequent mutations (APC or β-catenin mutations) [12]. Recently, the antiparasitic drug nitazoxanide (NTZ), approved by the US Food and Drug Administration (FDA) for use in humans, has been reported to antagonize Wnt/β-catenin signaling by abrogating β-catenin independent of GSK-3β and APC [13], indicating the potential therapeutic value of NTZ in APC-mutated or β-catenin-mutated CRC.

Farnesoid X receptor (FXR, encoded by NR1H4), a bile acid-activated nuclear receptor, regulates the homeostasis of lipid, cholesterol and glucose metabolism [14]. Mounting evidence supports a pivotal role for FXR in colorectal tumorigenesis. Diminished FXR is significantly related to late tumor stage and often predicts a poor prognosis [15]. Loss of FXR promoted intestinal inflammation and colon tumorigenesis [16]. Conversely, activation of intestinal FXR can suppress abnormal cell growth and curtail CRC progression [17]. Thus, targeting FXR and restoring its function might be an attractive tactic for CRC treatment.

Obeticholic acid (OCA) is a novel FXR agonist and a derivative of chenodeoxycholic acid (CDCA) and shows almost 100-fold greater potency than CDCA [18]. Importantly, OCA has been approved by the FDA for the treatment of primary biliary cholangitis [19]. Recent studies indicate that OCA shows a promising antitumor effect in cholangiocarcinoma [20] and HCC [21]. However, the complicated signaling network and tumor heterogeneity might hinder the effectiveness of FXR agonists in cancer treatment [22]. This highlights the importance of identifying drug combinations or novel chemicals with potency and specificity to enhance the antitumor effects of FXR activation. Herein, our study aimed to explore the antitumor effect of OCA in CRC and further identify a potential target to rationally design a combinational approach based on an FXR agonist to combat CRC.

## Materials and methods

### Cell cultures

Colon cancer cells SW403, SW480, DLD-1, HT-29, HCT116, and RKO (Shanghai Institute of Cell Biology, Chinese Academy of Sciences) were all routinely cultured in DMEM (Gibco BRL, Carlsbad, CA, USA) supplemented with 10% fetal bovine serum (Gibco BRL, Carlsbad, CA, USA) at 5% CO_2_ at 37 °C. Once the cell confluence reaches 70%, they were treated with various doses of OCA (Sellerk, Houston, TX, USA) for 48 h in the presence or absence of NZT (Sellerk, Houston, TX, USA). For ICG-001, cells were incubated in the presence of ICG-001 (10 μM, Sellerk, Houston, TX, USA) for 24 h along with OCA. OCA (5 mg) was reconstituted in 1.1887 mL DMSO to 10 mM. NTZ (5 mg) was reconstituted in 1.6272 mL DMSO to 10 mM. The final concentration of DMSO in control and experimental groups was maintained at less than 0.1% in all treatments.

### Lentiviral vectors and transfection

The phU6-EGFP-shRNA-FXR and phU6-EGFP-shRNA-β-catenin lentiviral vectors and their control vectors were commercially purchased from GeneChem Co., Ltd. (Shanghai, China). The transfection process is completed in accordance with the manufacturer’s instructions.

### Drug combination studies

For in vitro experiment, Cells per well were seeded in the 96-well plates at a density of 3 × 10^3^. The following day, the cells were treated with a single compound or with a combination of OCA and NTZ for 48 h. CCK8 kit was used to measure cell viability. Combination index (CI) and fraction affected (Fa) values were calculated using CompuSyn software. CI > 1, CI = 1, and CI < 1 indicate antagonism, addictive and synergy effect respectively.

For in vivo experiment, *Q* value method of Zhengjun jin was adopted [23]. *Q* value > 1.15 was synergistic; 0.85–1.15 was additive; <0.85 was antagonistic.

### CCK8, colony formation, cell cycle, and apoptosis assays

For CCK8 assay, cells were seeded into 96-well culture plates at 3000 cells/well for 48 h. Cell viability was checked by CCK-8 assay according to manufacturer’s protocol. Normalization was done to cells treated with DMSO as vehicle, which were defined as 100%. For colony formation assay, three hundred cells were seeded and cultured for 14 days. Colonies (≥50 cells/colony) were counted. For cell cycle determination, cells were seeded at a density of 1 × 10^5^ cells/well in 6‑well plates. Cells were collected and fixed in 75% freezing ethanol and kept overnight at 4 °C. After digesting with RNase A for 30 min at 37 °C, the cells were incubated with propidium iodide for 30 min shielded from light. The cell cycle was evaluated with flow cytometry (BD, Franklin Lakes, NJ, USA). For apoptosis assay, cells were seeded at a density of 1 × 10^5^ cells/well in 6‑well plates. Cells were labeled with Annexin V PE/7-AAD (BD Biosciences, Franklin Lakes, NJ, USA) according to the manufacturer’s protocol as previously described [24]. Each experiment was performed in triplicate.

### Transwell assays

Cell invasion was measured by using Transwell inserts (Corning, New York, NY, USA) with Matrigel (BD, Franklin Lakes, NJ, USA). The under chamber was filled with 600 μL of RPMI 1640 medium added with 20% FBS. The upper chamber filters were precoated with 50 µL of Matrigel and plated at 1 × 10^5^ cells per upper chamber. The cells were incubated at 37 °C for 48 h. After incubation, cells not through the aperturs on the upper surface of the Transwell inserts were got rid of with fresh PBS. The migratory or invading cells on the underside of the membrane were fixed with 4% paraformaldehyde and dyed with 1% crystal violet. The number of cells was counted in three randomly selected fields of fixed cells under an inverted microscope. Each experiment was repeated three times.

### Nude mouse xenograft assay

All animal experiments were conducted in line with the institutional guidelines, and was authorized by the Institutional Animal Care and Use Committee of the First Affiliated Hospital of Xi’an Jiaotong University. The female BALB/c-nude mice (5-week-old) were purchased from a corporation of Shanghai (SLAC Laboratory Animal Co, China). The mice were injected with 5 × 10^6^ colon cancer cells into the right flanks to construct xenograft tumor mice model. Once the size of xenograft tumors reached approximately 100 mm^3^, the nude mice were randomly divided into four subgroups and were administered by oral gavage of OCA (10 mg/kg/day) and NTZ (200 mg/kg/day) alone or in combination for consecutive 18 days. Tumor size was monitored using callipers every 3 days, and the tumor volume was measured the according to the formula (*a* × *b*^2^ × 0.5, *a*: length, *b*: width). After 18 days of drug administration, the mice were executed and the xenograft tumors were isolated and weighted.

### RNA isolation and real-time PCR

TRIzol® reagent (Invitrogen; Thermo Fisher Scientific, Inc.) was employed to extract total RNA from cells in accordance with the manufacturer’s instructions. The PrimeScript® RT Reagent kit (Takara Biotechnology Co., Ltd., Dalian, China) was employed to reverse transcription in the condition of 37 °C for 15 min and 85 °C for 5 s, followed by reserved at 4 °C. Real-time PCR was performed using SYBR Premix Ex Taq II (Takara Biotechnology Co., Ltd.) on a PCR Detection system (CFX96™, Bio-Rad Laboratories, Inc., Hercules, CA, USA). The qRT-PCR condition adopted two-step method: pre-denaturation at 95 °C for 30 s followed by forty cycles of denaturation at 95 °C for 5 s and extension at 60 °C for 1 min. The relative mRNA expression level was analyzed by using the 2^−ΔΔCq^ method [25]. The sequences of primers were summarized in Supplementary Table S1. Each experiment was repeated three times.

### Immunohistochemistry (IHC)

Fresh tissues were quickly fixed with 10% neutral formalin at room temperature overnight and embedded in paraffin. Afterwards, 4-μm tissue slides were prepared. The standard Streptavidin-Biotin Complex (SABC) method was adopted to perform IHC staining procedure. Primary antibodies were employed in the slides with the concentration of 1:100 overnight at 4 °C, and then biotinylated secondary antibody (Wuhan Boster Biological Technology, Ltd., Wuhan, China) was employed for 30 min at 37 °C. The images were collected by using a Nikon ECLIPSE Ti-S microscope mounted with a Nikon digital camera (Nikon Corporation), and all specimens were independently evaluated by two researchers.

### Total protein extraction and Western blot

The cells were lysed with RIPA buffer containing containing a protease inhibitor cocktail on ice for 40 min. The BCA method (Pierce; Thermo Fisher Scientific, Inc.) was used to quantify each sample. Separate the protein by SDS-PAGE and transfer the protein to activated polyvinylidene difluoride membranes. Following 5% skim milk, the membranes were incubated with primary antibodies followed by incubation with horseradish peroxidase (HRP)-conjugated secondary antibodies. The visualized signals of the bands were acquired by exposing to Chemiluminescent HRP Substrate (Millipore, Billerica, MA, USA) via protein imprinting imaging system and the protein expression was analyzed using Image software (National Institutes of Health, Bethesda, MD, USA). The detailed information regarding these antibodies was presented in Supplementary Table S2. Each experiment was repeated three times.

### Immunofluorescence (IF)

Cells were fixed with 4% paraformaldehyde for 20 min, and then punched with 0.2% Triton X-100 for 10 min. Block cells with 5% bovine serum albumin at room temperature for 30 min, and then incubate cells with primary antibody (anti-FXR with 1:100 dilution) at 4 °C overnight. The sections were washed three times with PBS for 10 min each time, and then incubated with Alexa Fluor 594-conjugated secondary antibody (1:400 dilution, Invitrogen, Carlsbad, CA, USA) for 1 h at room temperature and protected from light. The nucleus was stained with DAPI for 10 min. Finally, the sample was observed using a fluorescence microscope (Leica Microsystems, Heidelberg, Germany) to analyze the subcellular localization of FXR. Each experiment was repeated three times.

### Immunoprecipitation (IP) assay

After fresh cells were washed three times with cold PBS, they were collected by IP lysis. The bait protein antibody (anti-FXR antibody) is cross-linked with Amino-Link plus coupling resin (Pierce Co-IP kit, Rockford, IL, USA) at room temperature for 1–2 h according to the method of the manufacturer’s instructions. About 500 µg of protein lysate was added to resin-antibody complex and was shook slowly at 4 °C overnight. After the protein complex is eluted from resin, it is separated by western blotting after high temperature deformation. The target protein antibody (anti-β-catenin) was used to detect the interaction with the bait protein. IgG antibody was used as a negative control to exclude nonspecific binding. Each experiment was repeated three times.

### Quantitative chromatin immunoprecipitation (qChIP)

EZ-ChIP Kit (Millipore, Bedford, MA, USA) was used in fresh cells for ChIP experiments according to the method of the manufacturer’s instructions. Five microgram of anti-FXR antibody and 1 μg IgG negative control antibody were used to precipitate the chromatin-protein mixture, and finally amplified with specific primers (Supplementary Table S1) of the target fragment or endogenous noncoding region fragment and detected its expression using real-time quantitative PCR. The qRT-PCR condition adopted two-step method: pre-denaturation at 95 °C for 30 s followed by forty cycles of denaturation at 95 °C for 5 s and extension at 60 °C for 1 min. The formula for calculating the enrichment index was *E*^(Input Cq-ChIP Cq)^/*E*^(Input Cq-Control Cq)^. Each experiment was repeated three times.

### Statistical analysis

The differences among the experimental group and control group were compared by the Student’s *t*-test or one-way ANOVA. All statistical results were done using the SPSS statistical package (SPSS Inc., Chicago, IL, USA). *P* < 0.05 was considered as statistically significant.

## Results

### β-Catenin level determines the varying responses of colon cancer cells to OCA

**Fig. 1:**
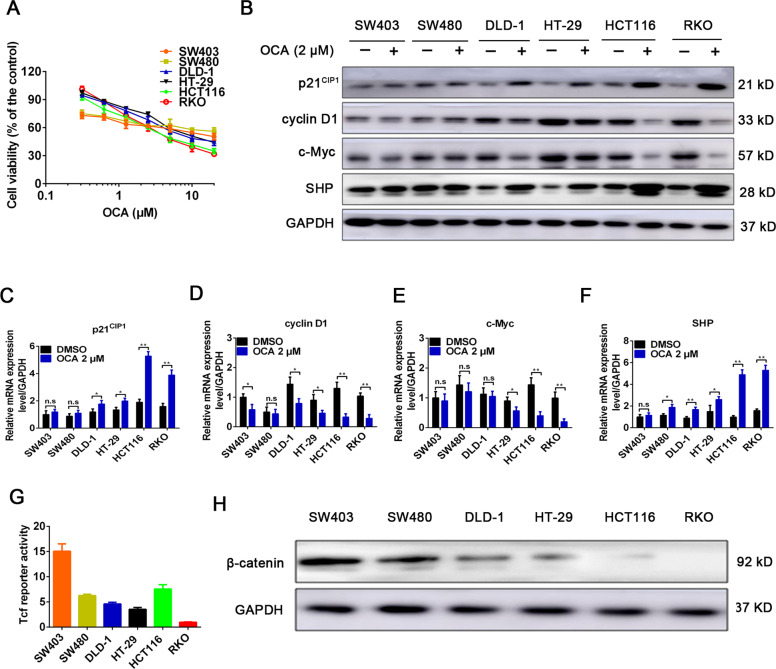
β-Catenin level determines the varying responses of colon cancer cells to OCA. **a** The effect of FXR agonist OCA on the viability of colon cancer cells SW403, SW480, DLD-1, HT-29, HCT116, and RKO detected by CCK8 assays. **b** The effect of OCA on the protein levels of p21^CIP1^, cyclin D1, c-Myc, and SHP in colon cancer cells detected by western blotting analysis. **c**–**f** The effect of OCA on the mRNA levels of p21^CIP1^, cyclin D1, c-Myc, and SHP in colon cancer cells detected by real-time PCR. **g** The effect of OCA on the luciferase activities of TOP/FOP-Flash reporter plasmid in colon cancer cells. **h** The expression of β-catenin in colon cancer cells detected by western blotting analysis. All data are the mean ± SD of three independent experiments. *P < 0.05, **P < 0.01.

**Fig. 2:**
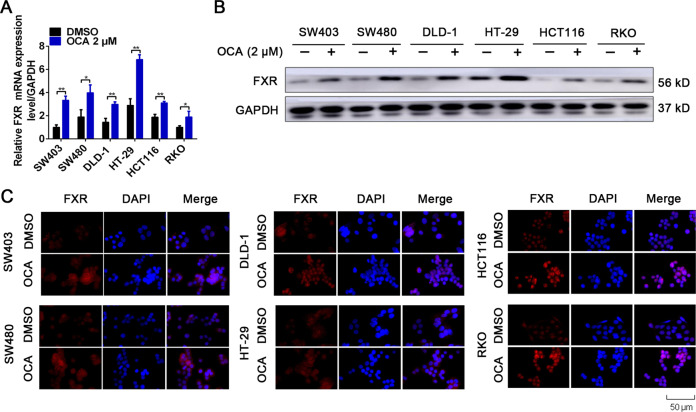
OCA induced FXR activation in colon cancer cells. **a** The effect of OCA on the mRNA level of FXR in colon cancer cells detected by real-time PCR. **b** The effect of OCA on the protein level of FXR in colon cancer cells detected by western blotting analysis. **c** The effect of OCA on the nuclear translocation of FXR detected by IF staining. Scale bars, 50 μm. All data are the mean ± SD of three independent experiments. *P < 0.05, **P < 0.01.

The effect of FXR activation by OCA on the growth of six colon cancer cells was assessed by CCK8 assays. The results showed that RKO and HCT116 cells showed the most sensitivity to OCA with IC50 values of 0.9211 and 0.8377 μM, respectively, whereas DLD-1 and HT-29 cells were moderately resistant to OCA with IC50 values of 2.044 and 2.993 μM, respectively (Fig. 1a). However, SW403 and SW480 cells showed resistance to OCA with IC50 values of 5.344 and 3.994 μM, respectively (Fig. 1a). SHP, the well-known target of FXR, has been proven to repress tumor growth by inducing apoptosis and cell cycle arrest [26]. As expected, after OCA exposure, RKO and HCT116 cells had dramatic changes in the mRNA and protein levels of p21^CIP1^, cyclin D1, c-Myc, and SHP, whereas DLD-1 and HT-29 cells showed moderate changes (Fig. 1b–f and Supplementary Fig. 1a–d). However, SW403 and SW480 cells failed to show these changes (Fig. 1b–f and Supplementary Fig. 1a–d). We next evaluated the levels of FXR in six colon cancer cells in response to OCA exposure. Despite the fact that OCA increased the levels of FXR in colon cancer cells (Fig. 2a, b and Supplementary Fig. 1f), no correlation between FXR levels and OCA sensitivity was observed. OCA performs its function by expediting FXR nuclear translocation and occupancy of its target genes [27]. The results of IF assays indicated that RKO and HCT116 cells after OCA exposure had dramatic nuclear localization of FXR, whereas the other four cell lines treated with OCA exhibited moderate or low nuclear localization of FXR (Fig. 2c). From these observations, we inferred that there might exist contributing factors that influence the nuclear localization of FXR induced by OCA and thereby affect the antitumor effect of OCA.

SW403, SW480, DLD-1, and HT-29 cells have APC mutations, and HCT116 cells β-catenin mutations, whereas RKO cells have no Wnt/β-catenin signaling-related mutations [28]. We reasonably questioned the correlation between the activity of Wnt/β-catenin signaling and OCA sensitivity. However, we did not observe a correlation between TCF/LEF transcriptional activities and OCA sensitivity (Fig. 1g). Intriguingly, RKO and HCT116 cells, which were sensitive to OCA expressed low or no levels of β-catenin, while colon cancer cells resistant to OCA harbored high levels of β-catenin (Fig. 1h and Supplementary Fig. 1e). Among them, SW403 and SW480 cells had the highest expression of β-catenin. We inferred that β-catenin level might affect the antitumor effects of OCA on colon cancer cells.

### Loss of β-catenin sensitizes colon cancer cells to the antitumor effect of an FXR agonist

**Fig. 3:**
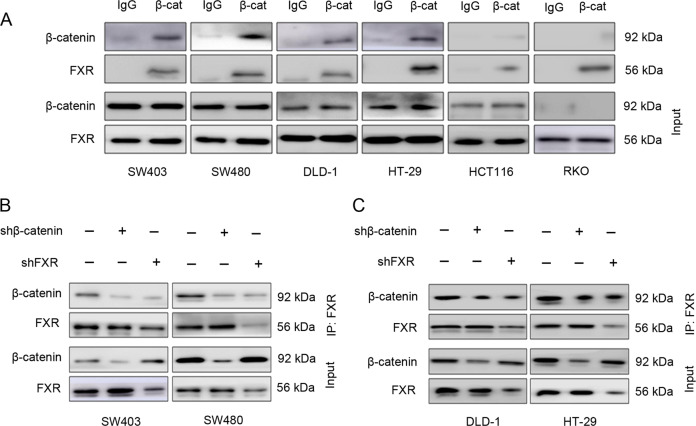
Identification of FXR/β-catenin complex in colon cancer cells. **a** The physical interaction between FXR and β-catenin in colon cancer cells. The cell lysates were subjected to IP assay with an anti-β-catenin antibody. **b** The depletion of β-catenin or FXR impaired the interaction of FXR and β-catenin in SW403 and SW480 cells. **c** The depletion of β-catenin or FXR impaired the interaction of FXR and β-catenin in DLD-1 and HT-29 cells.

We explored the detailed mechanism by which β-catenin regulates the antitumor effects of OCA. Deficiency of FXR resulted in sustained activation of the Wnt/β-catenin pathway [29]. Firstly, we supposed whether there exists any physical interaction between β-catenin and FXR. IP of lysates with a β-catenin antibody demonstrated that FXR and β-catenin interacted with each other in colon cancer cells (Fig. 3a); moreover, this interaction was abolished upon β-catenin or FXR depletion (Fig. 3b, c).

**Fig. 4:**
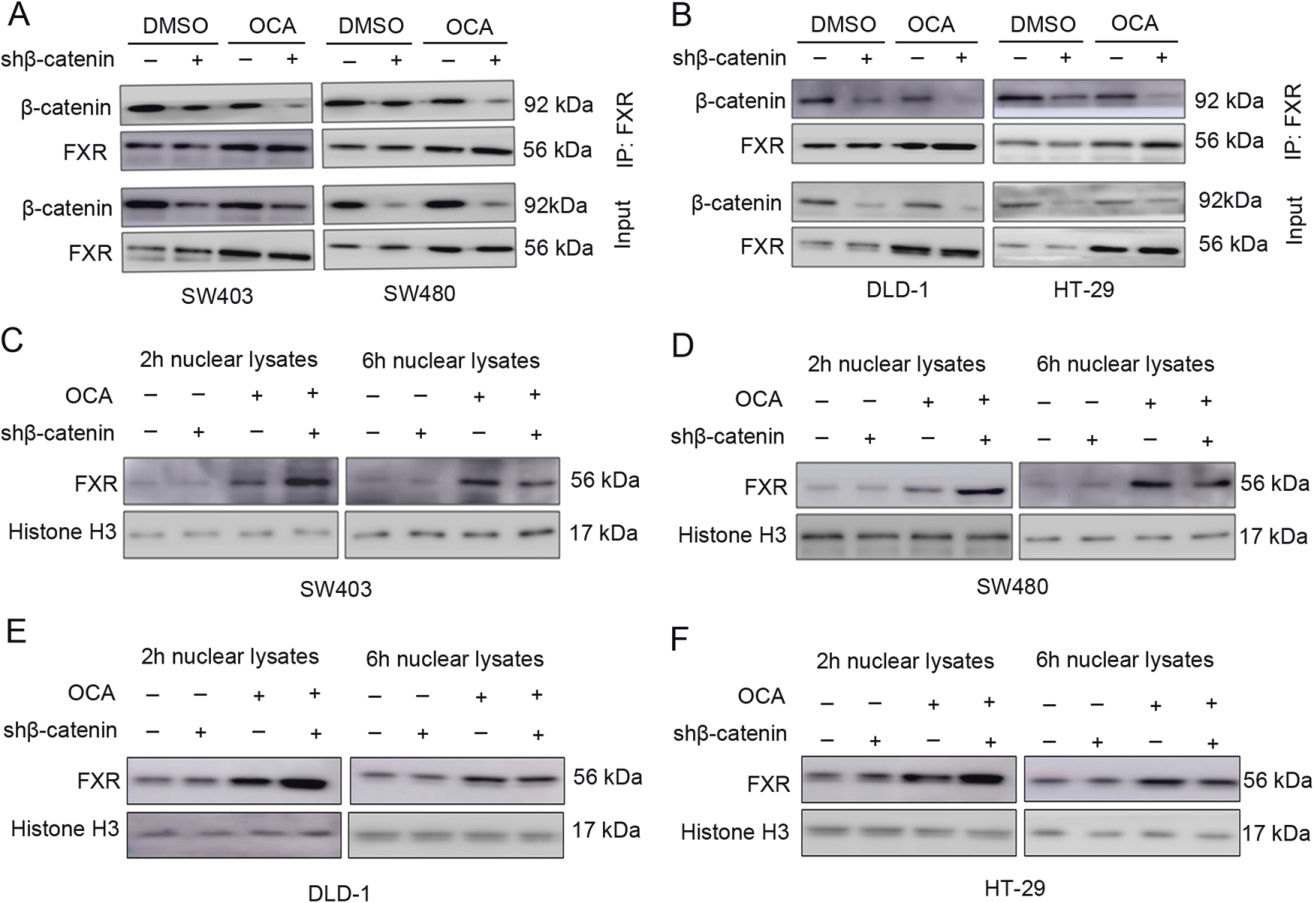
Depletion of β-catenin expedites FXR/β-catenin dissocation induced by OCA. **a** The effect of depleting β-catenin expression on the dissociation of FXR and β-catenin induced by OCA in SW403 and SW480 cells detected by IP assays. **b** The effect of depleting β-catenin expression on the dissociation of FXR and β-catenin induced by OCA in DLD-1 and HT-29 cells detected by IP assays. **c**–**f** The effect of depleting β-catenin expression on the nuclear translocation of FXR in colon cancer cells treated with OCA detected by western blotting analysis.

**Fig. 5:**
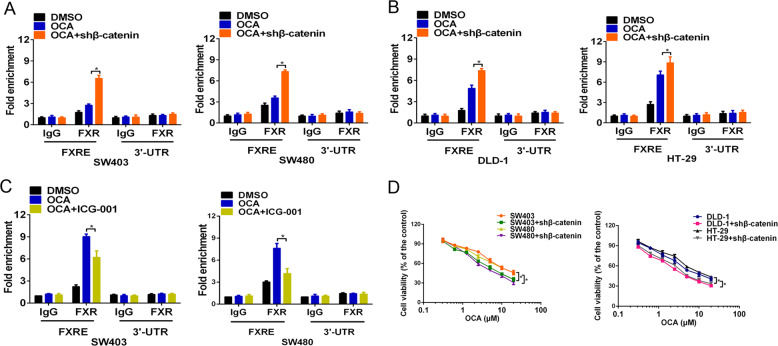
Depletion of β-catenin affects the binding of FXR to SHP promoter induced by OCA. **a**, **b** The effect of depleting β-catenin expression on the binding of FXR protein to the SHP promoter in colon cancer cells detected by the qChIP assays. **c** The effect of ICG-001, an antagonist of β-catenin/TCF4 complex, on the binding of FXR protein to the SHP promoter in colon cancer cells detected the qChIP assays. **d** The effect of depleting β-catenin expression on the viability of colon cancer cells detected by CCK8 assays. All data are the mean ± SD of three independent experiments. *P < 0.05, **P < 0.01.

We next hypothesized that β-catenin might affect FXR nuclear localization induced by OCA [30]. To confirm this notion, we evaluated the effect of depleting β-catenin expression on FXR nuclear localization of SW403, SW480, DLD-1, and HT-29 cells treated with OCA. The IP assay showed that the OCA treatment induced the dissociation of the FXR/β-catenin complex; notably, β-catenin depletion expanded this dissociation (Fig. 4a, b). Moreover, a marked elevation of nuclear FXR occurred in β-catenin-depleted cells as early as 2 h after OCA treatment, while a comparable elevation of nuclear FXR occurred in the control cells at 6 h (Fig. 4c–f), indicating that depletion of β-catenin accelerates FXR nuclear localization. As expected, depletion of β-catenin enhanced the binding of FXR to the SHP promoter after OCA treatment, as detected by the qChIP assay (Fig. 5a, b). Intriguingly, treatment with ICG-001, an antagonist of β-catenin/TCF4-mediated transcription, did not induce occupancy of FXR on the SHP promoter (Fig. 5c); conversely, ICG-001 suppressed this occupancy. This is likely due to ICG-001 elevating the free pool of β-catenin [22]. These observations indicated that β-catenin levels but not its transcriptional activity affected the antitumor effects of OCA. Importantly, depletion of β-catenin enhanced the inhibitory effect of OCA on the growth of SW403, SW480, DLD-1, and HT-29 cells (Fig. 5d). Taken together, these data strongly suggest that depletion of β-catenin makes FXR amenable to earlier activation and accelerates FXR nuclear translocation and occupancy of its target genes, thereby enhancing the antitumor effect of OCA.

### NTZ acts synergistically with OCA in colon cancer cells

The above observations inspired us to reason that modulation of β-catenin by chemical agents could enhance the antitumor effect of OCA on colon cancer cells. We assessed drug combination synergism in vitro by measuring the average combination index (CI) of OCA combined with the antiparasitic drug NTZ, which has been proven to abrogate β-catenin expression [31]. We first determined the IC50 values of NTZ in colon cancer cells. The results showed that NTZ inhibited the growth of SW403, SW480, DLD-1, and HT-29 cells with IC50 values of approximately 2.764, 2.294, 2.149, and 1.930 μM, respectively (Supplementary Fig. 2a). Moreover, NTZ caused dose-dependent repression of β-catenin expression in colon cancer cells (Supplementary Fig. 2c–f). Decreased β-catenin protein levels were followed by changes in the gene expression of the β-catenin downstream targets c-Myc and cyclin D1 (Supplementary Fig. 2b–f). These data indicated that NTZ inhibits the growth of colon cancer cells by abrogating β-catenin expression.

**Fig. 6:**
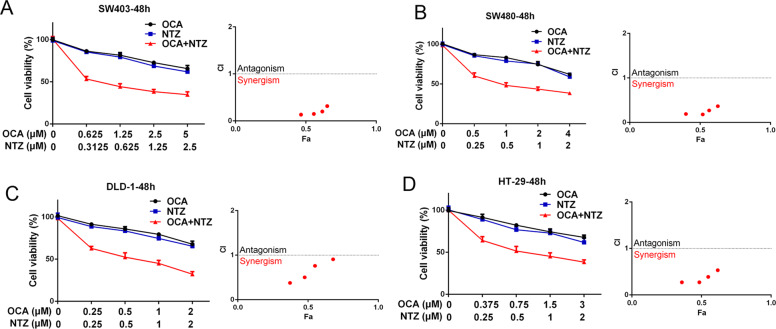
Drug combination screen identifies NTZ acting synergistically with OCA in colon cancer cells. **a**–**d** Sensitivity of SW403, SW480, DLD-1, and HT-29 cells to OCA, NTZ alone, or OCA plus NTZ. Survival fraction (left) and the CI (right) are shown for each of these four cell lines. Fa fraction affected. Error bars represent means ± SD.

The average CI values indicated that the antiparasitic drug NTZ had strong synergistic effects with OCA on SW403, SW480, DLD-1, and HT-29 cells (Fig. 6a–d). Altogether, this evidence suggests that NTZ might enhance the efficacy of OCA against CRC.

### OCA and NTZ synergistically inhibit the tumorigenic properties of colon cancer cells in vitro

**Fig. 7:**
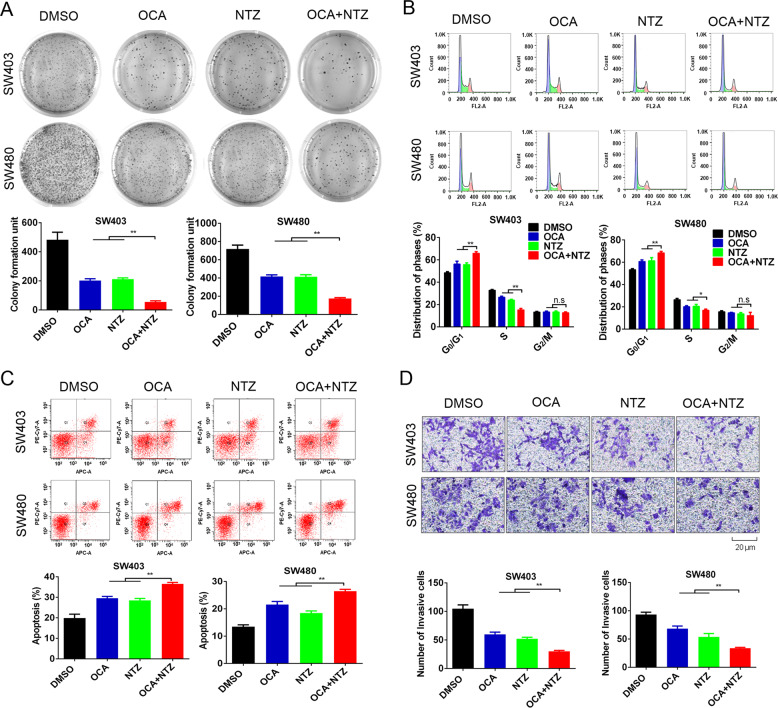
OCA and NTZ synergistically inhibited the tumorigenic properties of colon cancer cells in vitro. **a**, **b** The effect of OCA, NTZ alone, or OCA plus NTZ on colony formation and cell cycle distribution of SW403 and SW480 cells. **c**, **d** The effect of OCA, NTZ alone or OCA plus NTZ on the apoptosis and invasion of SW403 and SW480 cells. For SW403 cells, the concentrations of OCA and NTZ used were 1.25 μM and 0.625 μM, respectively. For SW480 cells, the concentrations of OCA and NTZ used were 1 μM and 0.5 μM, respectively. All data are the mean ± SD of three independent experiments. *P < 0.05, **P < 0.01.

By conducting a series of in vitro experiments, we evaluated whether NTZ enhanced the efficacy of OCA against CRC. SW403, SW480, DLD-1, and HT-29 cells were treated with OCA and NTZ alone or in combination. Compared to the single drug treatments, the combination of OCA and NTZ significantly repressed colony formation in colon cancer cells (Fig. 7a and Supplementary Fig. 3a). We further assessed the effects of combination therapy on cell cycle distribution and apoptosis by using flow cytometry. The combination of OCA and NTZ led to an increased percentage of cells in the G0/G1 phase and a decreased percentage in the S phase compared to the single drugs (Fig. 7b and Supplementary Fig. 3b). Moreover, an increase in the apoptosis rate was observed in cells treated with OCA and NTZ in combination compared to that in cells treated with the single drugs (Fig. 7c and Supplementary Fig. 3c). In addition, the combination of OCA and NTZ dramatically suppressed the invasive ability of cells relative to the single drug treatments (Fig. 7d and Supplementary Fig. 3d). Consistent with the observations above, the combination therapy of OCA and NTZ significantly elevated SHP expression and altered cell cycle-related and invasion-related proteins (Supplementary Fig. 4a–d). Altogether, this evidence suggests that NTZ enhances the efficacy of OCA in colon cancer cells.

### OCA and NTZ synergistically retard tumor growth in vivo

**Fig. 8:**
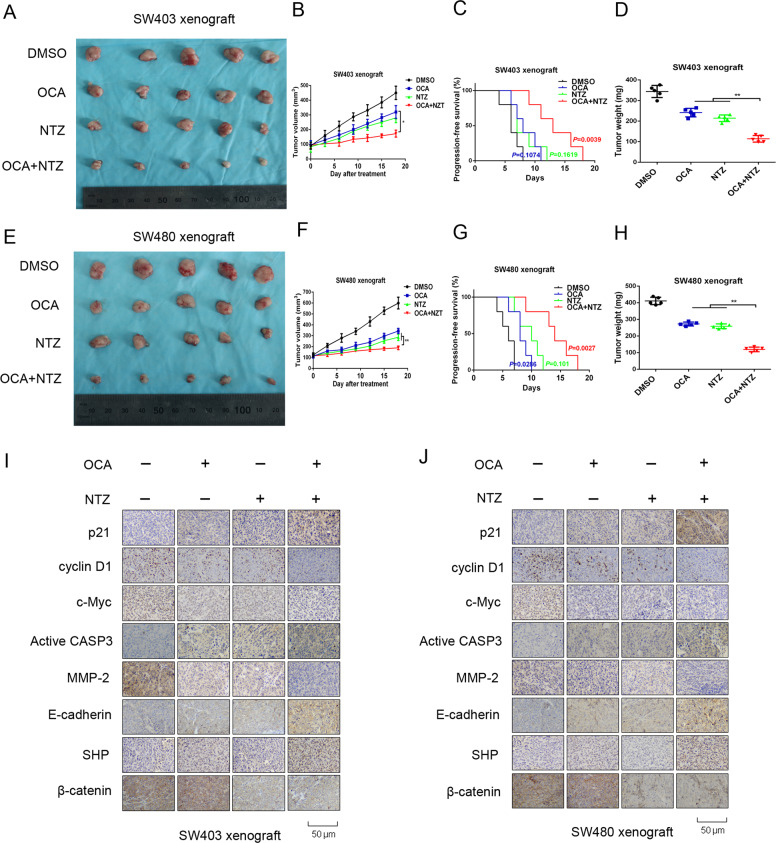
OCA and NTZ synergistically retarded tumor growth in vivo. **a** Schematic representation of the tumor xenografts formed by SW403 cells treated with OCA, NTZ alone or OCA plus NTZ. **b**, **d** Tumor growth curves (**b**) and tumor weights (**d**) for tumor xenograft formed by SW403 cells after OCA, NTZ alone or OCA plus NTZ treatment. **c** Cumulative incidence plot displaying the percentage of tumors in each treatment group that has doubled in volume as a function of time. P values were calculated with log-rank test. **e** Schematic representation of the tumor xenografts formed by SW403 cells treated with OCA, NTZ alone or OCA plus NTZ. **f**, **h** Tumor growth curves (**f**) and tumor weights (**h**) for tumor xenograft formed by SW403 cells after OCA, NTZ alone or OCA plus NTZ treatment. **g** Cumulative incidence plot displaying the percentage of tumors in each treatment group that has doubled in volume as a function of time. *P* values were calculated with log-rank test. **i**, **j** IHC staining for p21, cyclin D1, c-Myc, active caspase-3, MMP-2, E-cadherin, and SHP in tumor xenografts formed by SW403 (**i**) and SW480 (**j**) cells. *P < 0.05, **P < 0.01.

To validate the synergistic effects of NTZ and OCA in vivo, we generated xenograft mouse models by injecting colon cancer cells subcutaneously into nude mice. The xenograft tumors in the OCA-treated and NTZ-treated groups developed much more slowly and were smaller and lighter than those in the single drug-treated groups (Fig. 8a, b, d–f, h). According to *Q* value method of Zhengjun jin [23] (*Q* value > 1.15 was synergistic; 0.85–1.15 was additive; <0.85 was antagonistic), *Q* value for SW403 and SW480 xenografts is 1.19 and 1.22, respectively, indicating that combination of OCA and NTZ have synergistical effect. Importantly, the combination therapy significantly lengthened progression-free survival in relation to the single treatments (Fig. 8c, g). Mechanistically, the results from the IHC assay showed a stronger staining intensity of SHP, p21, E-cadherin, and active caspase-3 and a weaker staining of c-Myc, cyclin D1, and MMP-2 in xenograft tumors treated with both agents than in those treated with a single agent (Fig. 8i, j). In summary, these data suggested that OCA and NTZ can work synergistically to retard tumor growth in vivo.

## Discussion

Chemotherapy is the second major treatment type for CRC after surgical treatment, but there are many problems with chemotherapy, such as drug resistance and side effects [32]. Thus, more effective strategies and novel targets for chemotherapy in this malignancy are urgently required. The tumor suppressive role of FXR in colorectal carcinogenesis has inspired us to restore FXR expression as a novel therapeutic strategy [17]. However, the complicated signaling network and tumor heterogeneity might hinder the effectiveness of FXR agonists in cancer treatment [22]. Our study indicated that RKO and HCT116 cells that expressed no or low β-catenin were sensitive to OCA, whereas colon cancer cells harboring moderate or high levels of β-catenin were less sensitive or resistant to OCA. Notably, despite the increased FXR levels in all six colon cancer cells in response to OCA, only RKO and HCT116 cells had marked nuclear localization of FXR. We thus speculate that β-catenin might affect the localization of nuclear FXR induced by OCA, thereby antagonizing its antitumor effects. Subsequently, we identified a novel FXR/β-catenin complex in colon cancer cells, which was previously confirmed by the work of Thompson et al in primary hepatocytes [22]. The FXR/β-catenin complex might antagonize FXR nuclear localization and subsequent target gene transcription by FXR agonists [33]. As expected, depletion of β-catenin expedited FXR nuclear localization and enhanced its occupancy on the SHP promoter following OCA exposure. In HCC with β-catenin mutations [34], elevated β-catenin might sequester FXR and impair its ability to maintain bile acid homeostasis, which leads to intratumoral cholestasis. Intriguingly, activation of FXR blocked the Wnt/β-catenin signaling pathway in HCC [29], indicating that there might exist a reciprocal relationship between FXR and β-catenin. Collectively, these novel findings identify an unrecognized role of β-catenin in colorectal carcinogenesis via sequestration of FXR and repression of its activity.

Wnt/β-catenin signaling is one of the most promising targets for cancer chemotherapy, and a wide variety of Wnt inhibitors have been developed and are in the preclinical or clinical phase I stage [11]. Recently, the antiparasitic drug NTZ drew our attention. In contrast to previous Wnt inhibitors, NTZ antagonizes Wnt/β-catenin signaling independent of GSK-3β and APC [31]. NTZ increased the citrullination and degradation of β-catenin by stabilizing PAD2. Our study demonstrated that NTZ repressed the growth of colon cancer cells by abrogating β-catenin expression. Notably, NTZ depleted β-catenin in colon cancer cells with mutant APC or β-catenin, For cancer treatment, a new use for an “old drug” has many benefits without the limitation of unknown safety and toxicity profiles [35]. Hence, NTZ is expected to be a potential anticancer drug for cancer patients with Wnt pathway mutations.

Monotargeted therapies are new treatment options that have begun to enter cancer treatment. However, due to complicated signaling networks and tumor heterogeneity, the effectiveness of these drugs is still unsatisfactory [36]. Recently, combination therapy has gathered tremendous interest with its ability to enhance efficacy and reduce side effects; for example, GW4064 combined with acyclic retinoid (ACR) exerted synergistic inhibitory effects on the growth of HCC with lower doses of both agents [37]. The depletion of β-catenin in colorectal carcinogenesis expedites FXR nuclear localization and FXR occupancy of target genes and thus offers a novel therapeutic opportunity. Herein, we identified the antiparasitic drug NTZ with synergism with OCA against CRC. This synergistic effect of OCA and NTZ was verified in a series of in vitro and in vivo experiments. Furthermore, our study revealed that this synergistic effect was probably attributed to elevated SHP expression. SHP has also been shown to suppress tumor cell proliferation and invasion via transcriptional repression of cyclin D1 and Ccl2 expression [26, 38]. However, we cannot completely rule out that other possible mechanisms might also account for the synergistic effects of OCA and NTZ against CRC. The activation of FXR by GW4064 has been proven to antagonize Wnt/β-catenin signaling in HCC [29]. Hence, OCA may work synergistically with NTZ to repress the activity of Wnt/β-catenin signaling.

The present study demonstrates that β-catenin affects the antitumor effects of OCA on colon cancer cells. Mechanistically, we identified an FXR/β-catenin complex in colon cancer cells. Depletion of β-catenin accelerated the nuclear translocation of FXR and increased its occupancy of the SHP promoter in response to OCA treatment. Moreover, the combination of OCA plus the β-catenin inhibitor NTZ exerted synergistic tumor inhibition in CRC. Altogether, these findings offer useful evidence for the clinical use of FXR agonists combined with β-catenin inhibitors in combating CRC.

## Supplementary information

Supplemental figure 1

Supplemental figure 2

Supplemental figure 3

Supplemental figure 4

Supplemental legend

Supplemental table
