# Supplementary figures and images for: Synergistic tumor inhibition of colon cancer cells by nitazoxanide and obeticholic acid, a farnesoid X receptor ligand
Source: Cancer Gene Ther. 2020 Oct 13;28(6):590–601. doi: 10.1038/s41417-020-00239-8 (PMC8203497; doi:10.1038/s41417-020-00239-8)

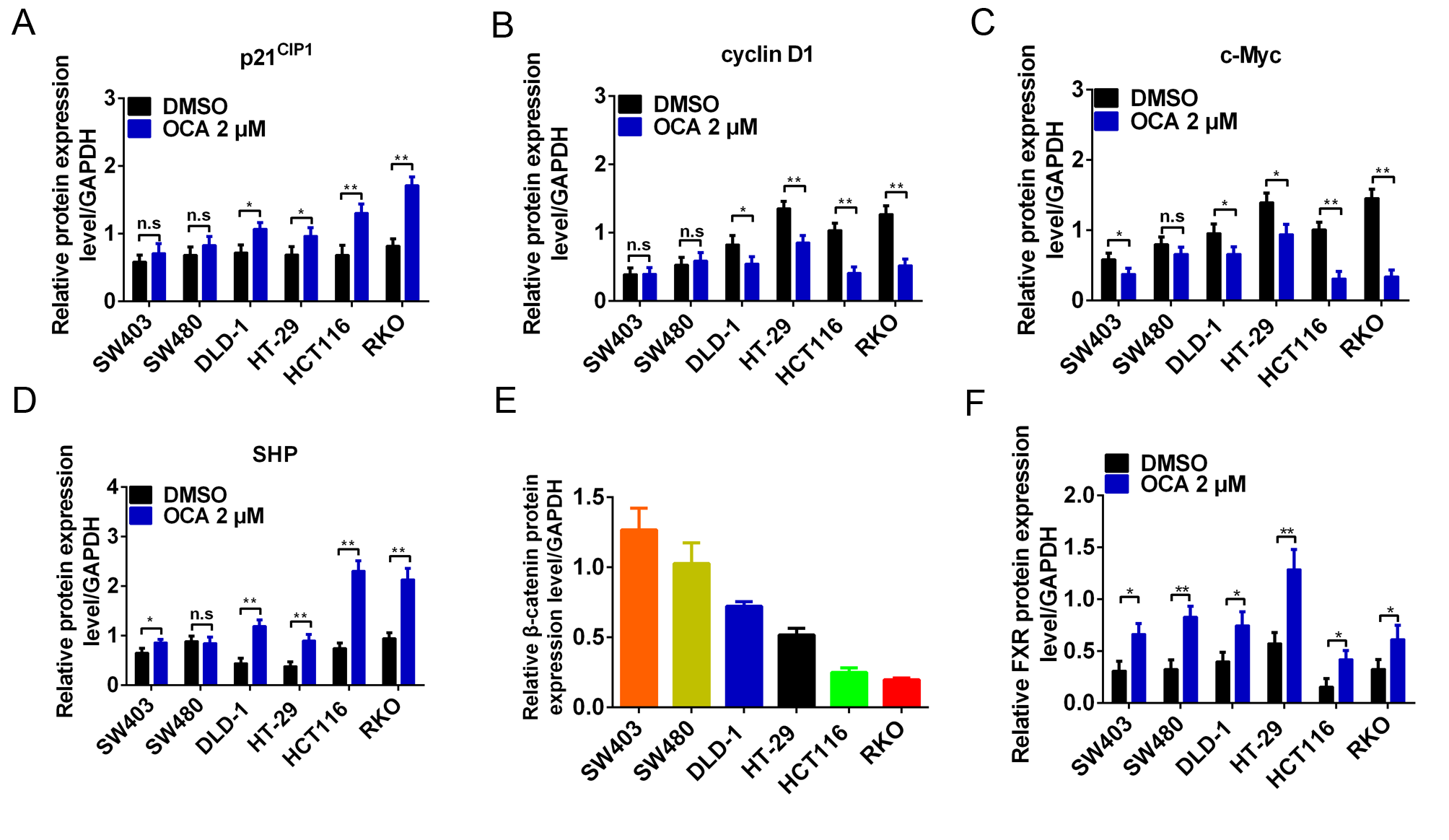

Supplement: Supplementary file 1 — Supplemental figure 1 [file 41417_2020_239_MOESM1_ESM.tif]

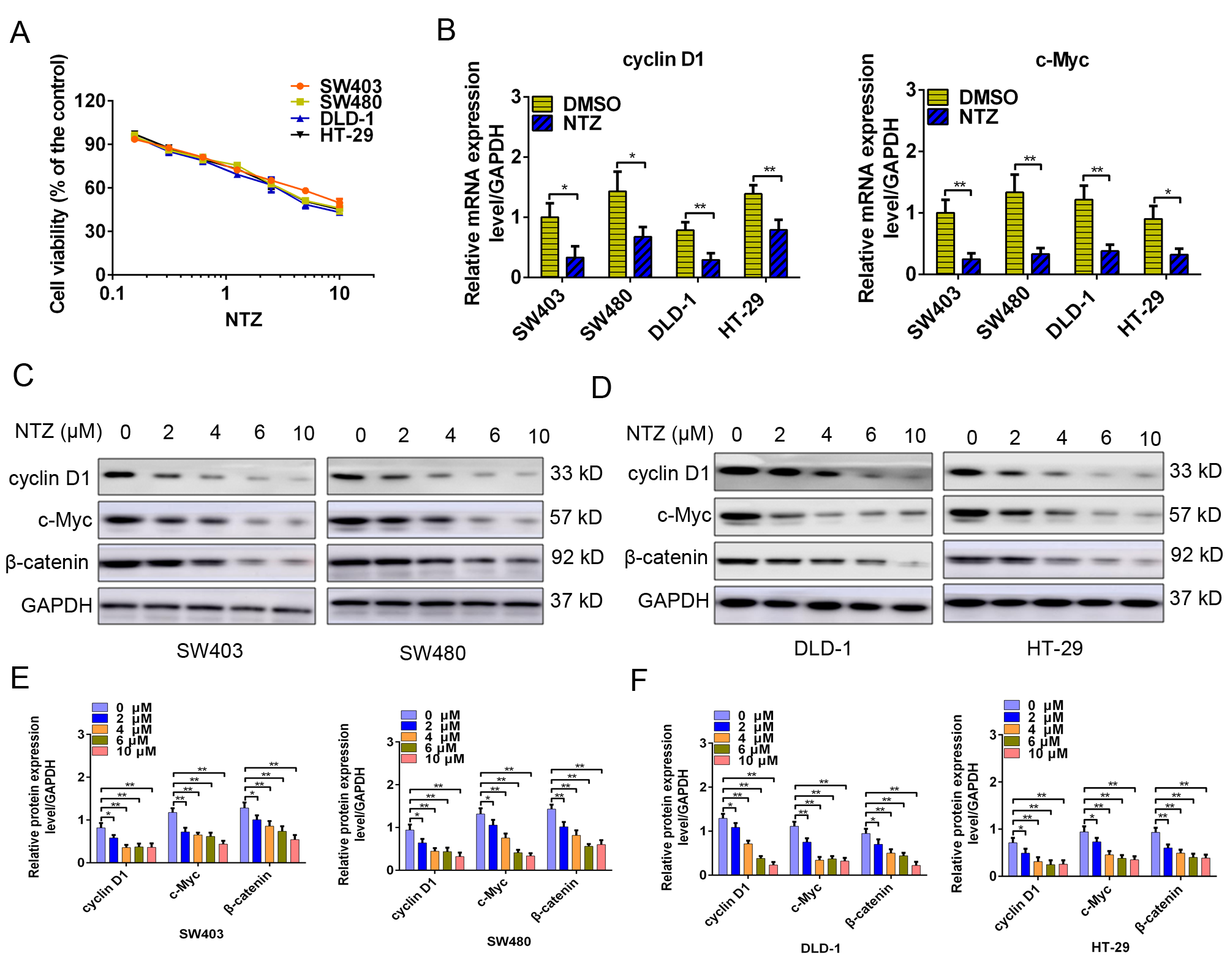

Supplement: Supplementary file 2 — Supplemental figure 2 [file 41417_2020_239_MOESM2_ESM.tif]

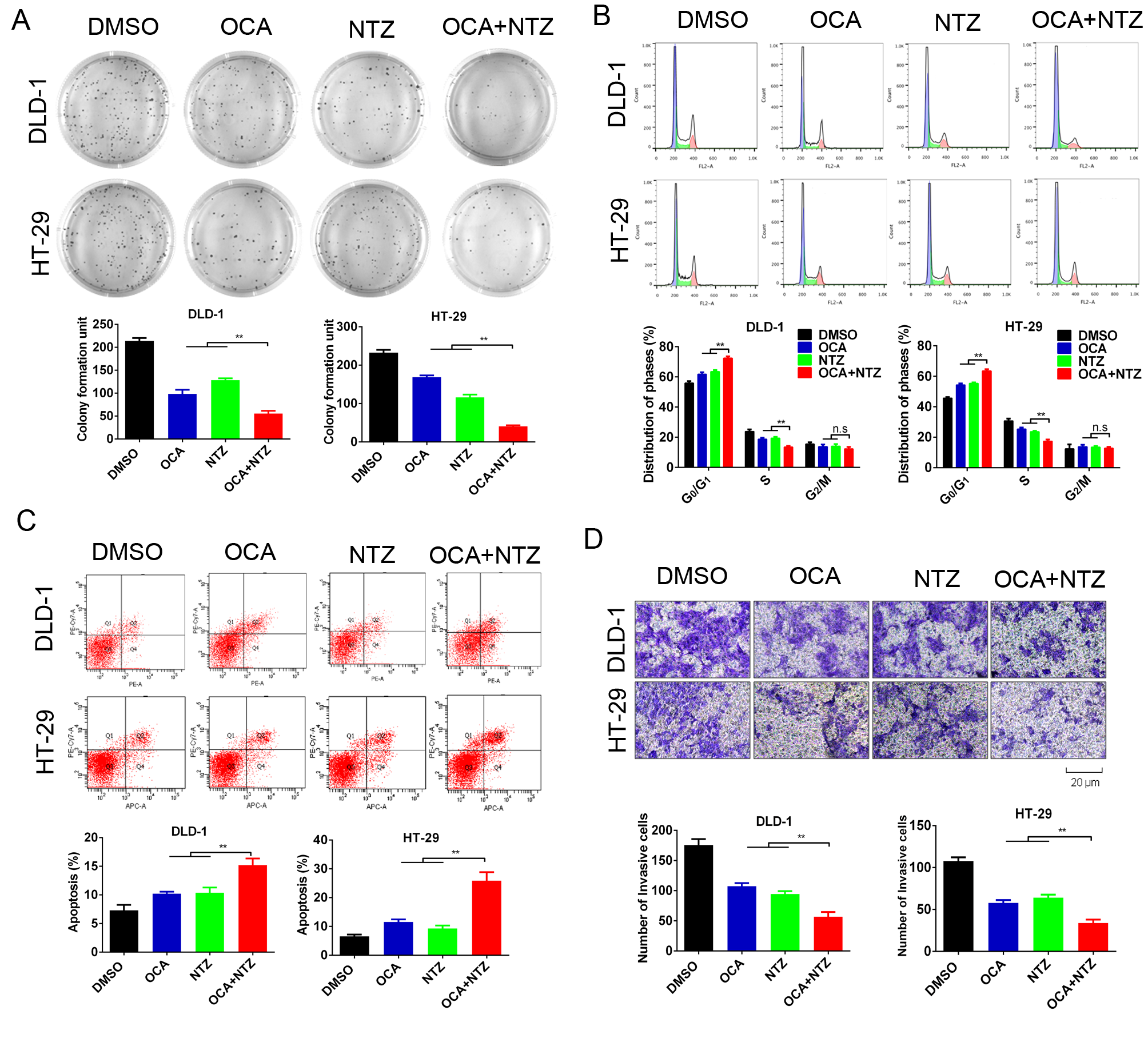

Supplement: Supplementary file 3 — Supplemental figure 3 [file 41417_2020_239_MOESM3_ESM.tif]

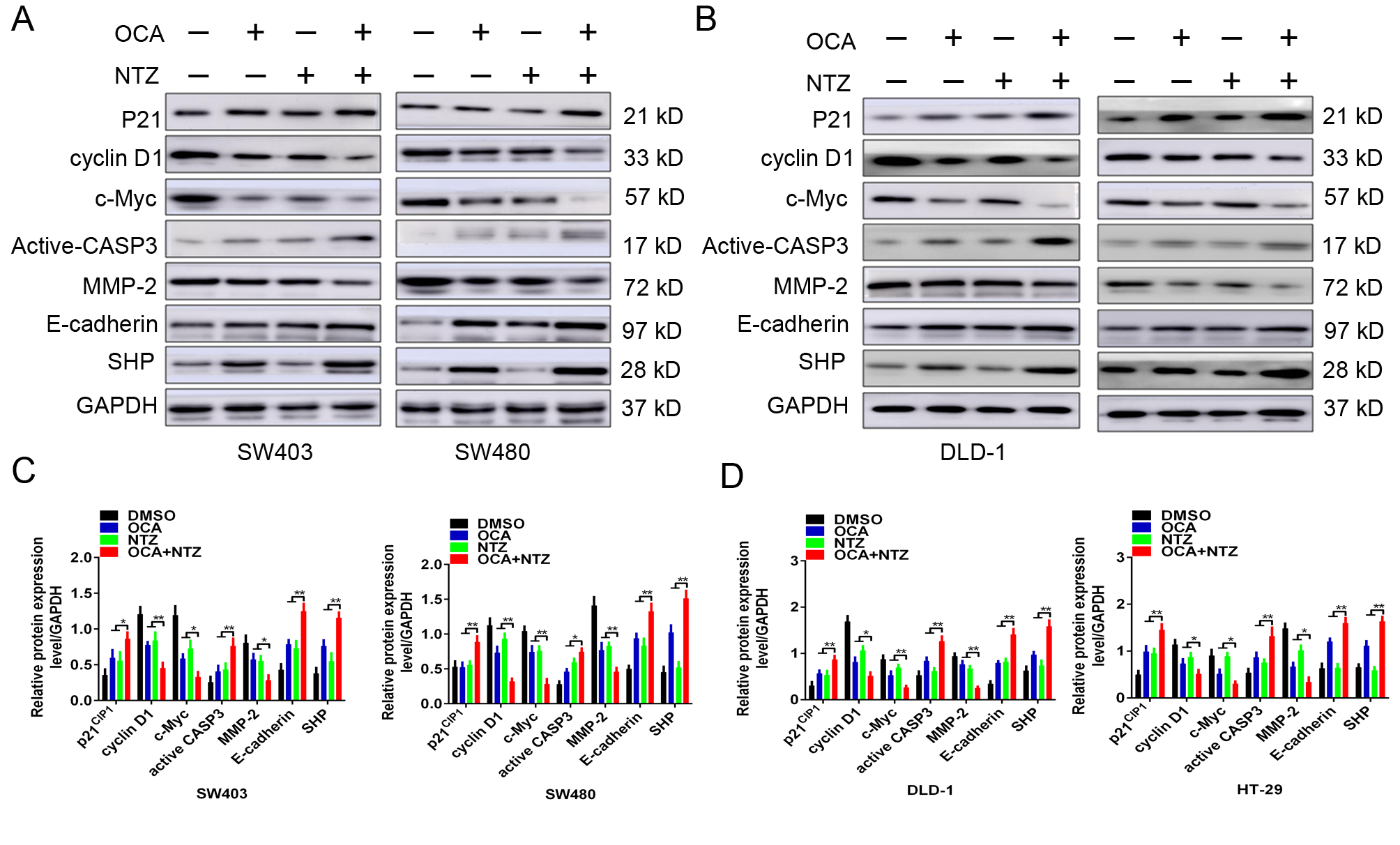

Supplement: Supplementary file 4 — Supplemental figure 4 [file 41417_2020_239_MOESM4_ESM.tif]
