# Supplementary material for: Synergistic tumor inhibition of colon cancer cells by nitazoxanide and obeticholic acid, a farnesoid X receptor ligand
Source: Cancer Gene Ther. 2020 Oct 13;28(6):590–601. doi: 10.1038/s41417-020-00239-8 (PMC8203497; doi:10.1038/s41417-020-00239-8)
Supplement: Supplementary file 5 — Supplemental legend [file 41417_2020_239_MOESM5_ESM.docx]

**Fig. 1 FXR agonist-resistant colon cancer cells harbor high levels of β-catenin**

**a-d** Quantitative analysis of p21^CIP1^, cyclin D1, c-Myc and SHP poteins in colon cancer cells detected by western blotting analysis. **e** Quantitative analysis of β-catenin protein in colon cancer cells detected by western blotting analysis. **f** Quantitative analysis of FXR protein in colon cancer cells treated with OCA detected by western blotting analysis. All data are the mean±SD of three independent experiments. **P*<0.05, ** *P*<0.01.

**Supplemental Fig. 2 NTZ inhibits the viability of colon cancer cells a** The effect of NTZ on the viability of colon cancer cells detected by CCK8 assays. **b** The effect of NTZ on the mRNA levels of cyclin D1 and c-Myc in colon cancer cells detected by Real-time PCR. **c, d** The effect of NTZ on the protein levels of cyclin D1, c-Myc and β-catenin in colon cancer cells detected by western blotting analysis. e, f Quantitative analysis of cyclin D1, c-Myc and β-catenin in colon cancer cells. All data are the mean±SD of three independent experiments. **P*<0.05, ** *P*<0.01.

**Supplemental Fig. 3 OCA and NTZ synergistically inhibit the tumorigenic properties of colon cancer cells in vitro** **a, b** The effect of OCA, NTZ alone or OCA plus NTZ on colony formation (**a**) and cell cycle distribution (**b**) of DLD-1 and HT-29 cells. **c, d** The effect of OCA, NTZ alone or OCA plus NTZ on the apoptosis (**c**) and invasion (**d**) of DLD-1 and HT-29 cells. For DLD-1 cells, the concentrations of OCA and NTZ used were 0.5 μM and 0.5 μM, respectively. For HT-29 cells, the concentrations of OCA and NTZ used were 0.75 μM and 0.5 μM, respectively. All data are the mean±SD of three independent experiments. **P*<0.05, ** *P*<0.01.

**Supplemental Fig. 4 OCA and NTZ synergistically regulated cell cycle-related and invasion-related proteins in colon cancer cells a, b** The effect of OCA, NTZ alone or OCA plus NTZ on the protein level of p21, cyclin D1, c-Myc, active caspase-3, MMP-2, E-cadherin and SHP in colon cancer cells detected by western blotting analysis. **c, d** Quantitative analysis of p21, cyclin D1, c-Myc, active caspase-3, MMP-2, E-cadherin and SHP in colon cancer cells. All data are the mean±SD of three independent experiments. **P*<0.05, ** *P*<0.01.
