# Supplementary material for: Synergistic tumor inhibition of colon cancer cells by nitazoxanide and obeticholic acid, a farnesoid X receptor ligand
Source: Cancer Gene Ther. 2020 Oct 13;28(6):590–601. doi: 10.1038/s41417-020-00239-8 (PMC8203497; doi:10.1038/s41417-020-00239-8)
Supplement: Supplementary file 6 — Supplemental table [file 41417_2020_239_MOESM6_ESM.docx]

Supplemental Table 1 Primer sequence

| Gene | NCBI accession | Sequence |
| --- | --- | --- |
| qRT-PCR |  |  |
| cyclin D1 | [NM_053056.3](https://www.ncbi.nlm.nih.gov/nuccore/NM_053056.3) | F: 5’- AAACAGATCATCCGCAAACAC-3’ |
|  |  | R: 5’-GTTGGGGCTCCTCAGGTTC-3’ |
| c-Myc | [NM_002467.6](https://www.ncbi.nlm.nih.gov/entrez/viewer.fcgi?db=nucleotide&id=1552482295) | F: 5’-CCTGGTGCTCCATGAGGAGA-3’ |
|  |  | R:5’-TCCAGCAGAAGGTGATCCAGAC-3’ |
| SHP | [NM_021969.3](https://www.ncbi.nlm.nih.gov/entrez/viewer.fcgi?db=nucleotide&id=1519241638) | F: 5’-CGATCCTCTTCAACCCAGATG-3’ |
|  |  | R: 5’-AGGGCTCCAAGACTTCACACA-3’ |
| GAPDH | [NM_001357943.2](https://www.ncbi.nlm.nih.gov/entrez/viewer.fcgi?db=nucleotide&id=1676440496) | F: 5’-TGCACCACCAACTGCTTAGC-3’ |
|  |  | R: 5’-GGCATGGACTGTGGTCATGAG-3’ |
| ChIP-qPCR |  |  |
| SHP | NG_012143.1 | F 5’-TGCCCTGGTACAGCCTGAGT-3’ |
|  |  | R 5’-ACACAGACATTGCCCCTGGC-3’ |

Supplemental Table 2 Antibodies

| Antibodies | Source | | Identifier |
| --- | --- | --- | --- |
| GAPDH | | Santa Cruz | Cat#sc-47724 |
| FXR | | Santa Cruz | Cat#sc-25309 |
| MMP-2 | | Abcam | Cat#ab92536 |
| SHP | | Abcam | Cat#ab96605 |
| CASP-3 | | Abcam | Cat#ab13847 |
| E-cadherin | | Cell Signaling Technology | Cat#14472 |
| cyclin D1 | | Cell Signaling Technology | Cat#55506 |
| c-Myc | | Cell Signaling Technology | Cat#18583 |
| p21^CIP1^ | | Cell Signaling Technology | Cat#2947 |
| β-catenin | | Cell Signaling Technology | Cat#8480 |
| Histone H3 | | Cell Signaling Technology | Cat#4499 |
